# Application Areas of Traditional Molecular Genetic Methods and NGS in relation to Hereditary Urological Cancer Diagnosis

**DOI:** 10.1155/2020/7363102

**Published:** 2020-06-17

**Authors:** Dmitry S. Mikhaylenko, Alexander S. Tanas, Dmitry V. Zaletaev, Marina V. Nemtsova

**Affiliations:** ^1^Laboratory of Epigenetics, Research Centre for Medical Genetics, Moscow 115522, Russia; ^2^Laboratory of Medical Genetics, I.M. Sechenov First Moscow State Medical University (Sechenov University), Moscow 119991, Russia

## Abstract

Next generation sequencing (NGS) is widely used for diagnosing hereditary cancer syndromes. Often, exome sequencing and extended gene panel approaches are the only means that can be used to detect a pathogenic germline mutation in the case of multiple primary tumors, early onset, a family history of cancer, or a lack of specific signs associated with a particular syndrome. Certain germline mutations of oncogenes and tumor suppressor genes that determine specific clinical phenotypes may occur in mutation hot spots. Diagnosis of such cases, which involve hereditary cancer, does not require NGS, but may be made using PCR and Sanger sequencing. Diagnostic criteria and professional community guidelines developed for hereditary cancers of particular organs should be followed when ordering molecular diagnostic tests for a patient. This review focuses on urological oncology associated with germline mutations. Clinical signs and genetic diagnostic laboratory tests for hereditary forms of renal cell cancer, prostate cancer, and bladder cancer are summarized. While exome sequencing, or, conversely, traditional molecular genetic methods are the procedure of choice in some cases, in most situations, sequencing of multigene panels that are specifically aimed at detecting germline mutations in early onset renal cancer, prostate cancer, and bladder cancer seems to be the basic solution for molecular genetic diagnosis of hereditary cancers.

## 1. Introduction

Diagnosis of renal cell cancer (RCC), prostate cancer (PC), and bladder cancer (BC) is an issue in the field of modern urological oncology because of their high incidence among malignant tumors and due to the social significance of these diseases [1]. As with cancers of other organs, solitary sporadic tumors that occur with advancing age account for a majority of urological oncology cases. Only 1% to 3% of these cases can be considered manifestations of hereditary cancer syndromes due to germline mutations. However, in many cases, hereditary forms of RCC, PC, and BC are associated with early onset, multiplicity of lesions, and specific nonurological signs, which make identification of germline mutations crucial for final diagnosis [2, 3]. Some hereditary urological cancer syndromes are monogenic diseases caused by point mutations of a single gene, and in some instances, common point mutations observed in a few exons can be diagnosed using relatively inexpensive, routine molecular genetic tests, such as polymerase chain reaction (PCR), multiplex ligation-dependent probe amplification (MLPA), and Sanger sequencing [4]. However, several new causative genes of hereditary urological cancer syndrome caused by germline mutations have recently been discovered via next generation sequencing (NGS) of the genomes and exomes of cancer patients. NGS has shown potential as a useful diagnostic technique when a multiexon candidate gene or several candidate genes must be examined to identify an underlying mutation [5, 6]. This review characterizes hereditary forms of RCC, PC, and BC (see Table 1) and suggests genetic diagnostic methods for these cases, including those for which balanced application of routine tests is justified and those for which NGS is indicated.

## 2. Clinical and Genetic Characteristics of Hereditary Forms of Renal Cancer

### 2.1. Von Hippel–Lindau Syndrome

Von Hippel–Lindau (VHL) syndrome (OMIM 193300) is an autosomal dominant hereditary cancer syndrome that occurs at a frequency of one per 39,000 to 90,000 newborns in different populations, with a penetrance >80% at the age of 60 [7]. The most common tumors are clear cell RCC (often multifocal and/or bilateral), renal cysts, hemangioblastomas of the central nervous system, retinal angiomas, and pheochromocytoma, whereas neuroendocrine tumors and cysts of the pancreas and endolymphatic sac tumors of the inner ear are far less common [8]. VHL syndrome is caused by mutation of the *VHL* tumor suppressor gene, which maps to chromosome 3p25 and has three exons and encodes a protein containing 213 amino acid residues. VHL normally binds to CUL2, RBX1, and elongins B and C to produce a multiprotein complex that promotes ubiquitin-dependent degradation of hypoxia-inducible factors 1/2*α* (HIF1/2*α*) [9]. Genotype-phenotype correlations are characteristic of *VHL* mutations in VHL syndrome, which is classified into type 1 (without pheochromocytoma but with high risk for clear cell RCC) and type 2 (with pheochromocytoma). Type 1 VHL syndrome is associated with frameshifts, nonsense mutations, and missense mutations that prevent the production of mature VHL protein. By contrast, type 2 VHL syndrome is associated with point missense mutations that cluster in regions encoding HIF and the elongin C binding sites of the VHL protein [10, 11]. An example of VHL syndrome demonstrates that identification of a causative pathological germline mutation can affect treatment decision. In sporadic kidney tumors, the primary tumor is removed after the completion of diagnostic tests and determination of disease stage. Because the risk of developing multiple tumors, including those in the contralateral kidney, with VHL syndrome is quite high, patients with VHL syndrome confirmed by molecular genetic testing are treated by removing the primary tumor via nephrectomy as soon as the tumor reaches 3 cm in the largest dimension along with certain contraindications [12, 13]. However, early metastasis is possible in other hereditary RCC forms, warranting surgery immediately after diagnosis. For example, type II papillary RCC in hereditary leiomyomatosis and RCC (described in the next section of this review) often develops as a solitary unilateral tumor but is characterized by rapid progression [14]. The accumulation of HIF in the cell and, in particular, of its HIF-2*α* isoform with oncogenic properties introduces the possibility of therapeutic inhibition of the intermediate pathogenetic pathway triggered by inactivation of *VHL*. Small synthetic inhibitors have been developed that block the heterodimerization of HIF-2*α* with HIF-1*β* and their DNA binding, thereby disrupting the activation of HIF target genes, as well as drugs that promote VHL-independent HIF degradation. These drugs (panobinostat, entinostat, vorinostat, bortezomib, and others) are used in clinical trials along with other types of targeted therapy in patients with metastatic clear cell RCC [15].

### 2.2. Hereditary Leiomyomatosis and RCC (HLRCC)

HLRCC (OMIM 150800) is associated with multiple leiomyomas (leiomyosarcomas in some cases) of the skin and uterus and RCC and identified in 25% of HLRCC patients. It is sometimes combined with renal cysts. Renal malignancies in this familial cancer syndrome are often type II papillary carcinomas [16]. Mutations of the fumarate hydratase (*FH*) tumor suppressor gene are responsible for HLRCC. *FH* is located on chromosome 1q42 and encodes an enzyme involved in the Krebs cycle. Missense mutations account for ∼90% of all relevant mutations, with no distinct hot spots identified in most populations worldwide (although in some European populations, hot spot mutations were identified in codon 190) [14]. Minor forms of HLRCC syndrome have been described. For example, a combination of type II papillary RCC and hemangioblastomas was observed in a patient with a 1.4 Mb deletion, which affected region 1q43, including nine genes in addition to *FH* [17]. It is worth noting that the role of germline *FH* mutations in carcinogenesis is not limited to HLRCC. A case of leiomyomatosis in combination with pheochromocytoma [18] was described, and *FH* was included in a panel of 10 genes to search for germline pathogenic variants in familial pheochromocytoma [19].

### 2.3. Birt–Hogg–Dube Syndrome (BHDS)

BHDS (OMIM 135150) is an autosomal dominant syndrome, a major manifestation of which includes multiple fibrofolliculomas. The presence of at least 10 characteristic skin neoplasms, including one histologically identified fibrofolliculoma, and a family history of the disease constitute the minimal diagnostic criteria potentially leading to a preliminary diagnosis of BHDS. Renal tumors, which develop in 35% of BHDS patients, are usually multifocal and bilateral and belong to different pathomorphological types, with chromophobe carcinomas and hybrid oncocytic/chromophobe tumors being the most common [20, 21]. BHDS is caused by germline mutations of folliculin (*FLCN*), a tumor suppressor gene located on chromosome 17p11.2. Germline *FLCN* mutations are predominantly loss-of-function mutations, such as frameshifts due to insertions, deletions, and duplications, as well as complex nonsense and splice-site mutations. Missense mutations were identified only in single cases [22, 23].

### 2.4. Hereditary Papillary Renal Carcinoma (HPRC) Type I

HPRC type I (OMIM 605074) is an autosomal dominant disease associated with the development of type I papillary RCCs, which are often multifocal and bilateral. Although inactivation of a tumor suppressor gene is responsible for the three hereditary RCC forms described, germline activating mutations of *MET*, an oncogene located on chromosome 7q31, causes HPRC [4]. *MET* germline mutations, observed in HPRC, lead to constitutive activation of the cytoplasmic domain of the receptor and stimulate cell division, constituting the main event in the carcinogenesis of papillary carcinomas in HPRC. Because *MET* activation is a key event in the HPRC pathogenesis, its therapeutic potential has been suggested for targeted therapy with MET inhibitors (i.e., foretinib, tivantinib, and volitinib) in metastatic HPRC. Studies of cabozantinib and the multikinase inhibitor crizotinib efficacy for papillary RCC treatment are also being carried out [24, 25]. The targeted MET inhibitor savolitinib led to a four-fold increase in relapse-free survival in papillary RCC with *MET* mutation [26]. Additionally, promising results have been reported for foretinib for treating metastatic HPRC in 10 patients, with treatment efficacy comparable with or even higher than that in sporadic type I papillary renal cancer. Therefore, MET inhibitors, especially foretinib, might be applicable for targeted therapy of HPRC [27].

### 2.5. *PBRM1* Mutations in Renal Cell Carcinoma

Exome sequencing in sporadic clear cell RCC identified *PBRM1*, a tumor suppressor gene, as the gene second most often altered by somatic point mutations: 38% of cases, trailing only *VHL* (50–60% of cases) [28]. A cohort of patients with suspected hereditary clear cell RCC and for whom VHL syndrome was excluded was screened for germline *PBRM1* mutations. Germline *PBRM1* mutations proved to be rare, with the inactivating germline mutation c.3998_4005del identified as the cause of the disease in only one of 35 unrelated candidate families [29].

### 2.6. The BAP1 Tumor Predisposition Syndrome

According to NGS-based studies, somatic mutations of *BAP1* are found in >10% of clear cell RCCs. *BAP1* encodes a deubiquitinating hydrolase that interacts with the tumor suppressor protein BRCA1. Additionally, *BAP1* is considered a tumor suppressor gene, although its functions are still being investigated. Data related to >180 families with germline *BAP1* mutations have been collected in recent years, enabling identification of target organs, tumor types, and description of the BAP1 tumor predisposition syndrome (BAP1-TPDS, OMIM 614327) as a new hereditary cancer syndrome caused by *BAP1* mutations. BAP1-TPDS is mostly associated with mesotheliomas, as well as cutaneous and uveal melanomas, with some patients developing RCC [30].

### 2.7. Hereditary Pheochromocytoma-Paraganglioma

Hereditary pheochromocytoma-paraganglioma is another rare hereditary cancer syndrome associated with RCC. Paragangliomas occur in 1 of 300,000 people, of which ∼25% are associated with germline mutations (i.e., the frequency of the syndrome is ∼1 : 1.2 million). This syndrome is caused by germline inactivating mutations of the tumor suppressor genes *SDHA*, *SDHB*, *SDHC*, *SDHD*, and *SDHAF2*, which encode subunits of the mitochondrial enzyme complex succinate dehydrogenase. In some cases, various pathomorphological types of RCCs caused mostly by *SDHB* and *SDHD* mutations might develop [31, 32]. Paragangliomas caused by germline mutations of *SDHB* can develop not only in the kidneys but also in the bladder. Lack of the SDHB protein according to immunohistochemical (IHC) analysis can be used as a screening test for possible *SDHB* mutation [33].

### 2.8. Tuberous Sclerosis

Tuberous sclerosis is a multisystem disease characterized by autosomal dominant inheritance and an incidence of one in 6,000 to 10,000 newborns. The disease exemplifies hereditary renal tumors with genetic heterogeneity and two multiexon candidate genes. Clinical signs of tuberous sclerosis include hypopigmented macules in 95% of cases and angiofibromas in 40% to 90% of cases. Multiple renal angiomyolipomas develop in 80% of tuberous sclerosis patients [34]. Germline mutations underlying tuberous sclerosis affect the tumor suppressor genes TSC complex subunit (*TSC*)*1* and *TSC2*, which encode hamartin and tuberin, respectively [35].

## 3. Methodological Approaches to Diagnosing Hereditary Forms of Kidney Cancer

The mission of the oncologist and geneticist in cases of suspected hereditary RCC is to refer the patient to molecular genetic testing to determine the most likely form of RCC and the candidate genes in which to search for a germline mutation. The choice of method for detecting mutations and the testing itself remain with the laboratory molecular diagnostics specialists. However, the choice of coding sequences of genes that should be investigated and, therefore, the choice of mutation analysis method only partially depend on the clinical picture of the disease. Most hereditary forms of RCC listed in the previous section present a characteristic clinical picture and can be considered as monogenic diseases according to the classical manifestation of the syndrome. In such cases, it is possible to search for mutations in only one candidate gene. In particular, in the classic form of VHL syndrome, diagnosis requires PCR amplification and sequencing of the three *VHL* exons (i.e., three PCR and six Sanger sequencing reactions per sample, which together with the intermediate steps of the analysis is feasible within 2 working days). Such analysis of point mutations allows identification of the cause of the disease in 85% of families with VHL syndrome [36–38]. In cases of negative results, partial deletions are additionally sought via MLPA or real-time PCR [39, 40]. However, again using VHL syndrome as an example, pitfalls of traditional molecular genetic methods also exist. For example, a 2C subtype of the syndrome appears only as adrenal pheochromocytoma and requires differential diagnosis with other hereditary cancer syndromes, such as hereditary pheochromocytoma-paraganglioma and type 2 multiple endocrine neoplasia [31, 41, 42]. In this case, it is necessary to examine a panel of six candidate genes. Moreover, although *VHL* and *RET* mutations can be detected by sequencing several PCR products, complementation of the testing area with SDH family genes immediately transfers these diagnostics to the category of tasks that have to be solved not by traditional methods of PCR and Sanger sequencing but by the multigene panel NGS [43].

Until recently, diagnosis of other monogenic forms of RCC was also carried out by PCR and Sanger sequencing because it was a question of sequencing no more than 10 different PCR products. In particular, direct DNA diagnosis of HPRC is based on the identification of missense mutations in *MET* exons 15 through 21, which encode the cytoplasmic domain of the receptor (7 PCR products) [12, 44]. PCR amplification and Sanger sequencing of all 10 *FH* exons enable DNA diagnosis of HLRCC [45]. Genetic laboratory diagnosis of BHDS is based on testing for *FLCN* mutations via PCR amplification and Sanger sequencing of polypeptide-coding exons 4 through 14. Note that the С_8_ mononucleotide tract of exon 11 is a mutation hot spot of *FLCN*, with germline single-nucleotide deletions/insertions in this tract found in ∼25% to 50% of affected families (i.e., exon 11 is expediently tested in the search for the underlying mutation) [46, 47]. In addition to testing for point mutations in *FLCN* exons, MLPA is performed to detect deletions, with their frequency in the promoter region of *FLCN* reported as higher than that in the coding region. Direct sequencing combined with MLPA increased the clinical sensitivity of molecular genetic testing from 80% to 95% of BHDS families [48], whereas a lower proportion of patients with verified mutations was reported by other studies [49].

However, in recent years, the search for germline mutations in *MET*, *FLCN*, and *FH* has been increasingly performed using NGS of multigene panels, specifically the TruSight Cancer Panel (Illumina), which includes 94 main oncogenes and tumor suppressor genes [50]. This is due to a decrease in the cost and time of performing NGS tests that has occurred in the previous 10 years, as well as to the similarity of the symptoms and the histological variants of tumors (see Table 1) observed in different forms of hereditary RCC. Basically, Illumina or Ion Torrent platforms with x20–50 reading depths are used to detect germline mutations [51, 52]. Various reviews in the previous 15 years have focused on comparing equipment cost and productivity, the sequencing price (including the cost of consumables), the percentage of errors in sequenced reads, and other technical characteristics of various NGS platforms [53, 54]. We note that in many respects, the price of the test and its execution time depend on the capacity of the chips and barcoding used, the frequency of sequencer usage per week (i.e., actually on the number of sequenced libraries of the same type), and the experience of the laboratory specialists in annotating cancer-related mutations.

Interestingly, the introduction of NGS in studies of the genomes of sporadic tumors and the search for somatic mutations sometimes provide unexpected results relevant to the diagnosis of hereditary cancer syndromes. In one study, *MET* mutations were detected in type I (presumably sporadic) papillary renal carcinomas, with three of the 17 mutations proving to be germline rather than somatic [55]. This finding indicated that testing for germline *MET* mutations was justified for younger patients with papillary renal carcinoma type I, even in the absence of multiple primary lesions in the kidneys. The sequencing of multigene panels is especially justified for mutation screening in genes comprising tens of exons. *PBRM1* has 10-fold more exons than *VHL* and lacks mutation hot spots, thereby warranting the use of NGS to search for mutations. Similar to *PBRM1*, *ВАР1* contains 17 exons and lacks recurrent mutations, warranting the utilization of NGS as the optimal technique and through which the pathological mutation might be identified during BAP1-TPDS diagnosis [30]. As noted, all genes of the SDH family must often be checked for mutations in order to diagnose hereditary pheochromocytoma-paraganglioma; therefore, exome sequencing or testing via proper gene panels is the appropriate method for direct DNA diagnosis of this syndrome [43]. *TSC1* contains 23 exons, and *TSC2* contains 42, with the relevant mutations distributed throughout the coding regions in a regular manner and including nonsense, missense, and splice-site mutations, as well as short insertions/deletions and extended deletions. Therefore, because these genes are large in size and lack mutation hot spots, NGS is warranted as the main method for potentially identifying causes of this syndrome. When confronted with negative sequencing results, MLPA is employed for expedient testing of extended deletions or duplications. A combination of these methods enables effective detection of germline mutations of *TSC1* and *TSC2* in 80% to 90% of tuberous sclerosis patients [56, 57].

At the same time, the larger the multigene panel, the more increased the number of identified genetic variants potentially difficult to classify in terms of clinical relevance, with this problem even more pronounced for exome sequencing. The guidelines of professional cancer associations do not currently include exome sequencing as an element of the diagnostic algorithms for RC, PC, or BC; therefore, it should be considered as an additional test with limited diagnostic value.

Finally, in the absence of criteria for known hereditary cancer syndromes but with a family history of the disease, early manifestation, and/or multiple tumors, the use of exome sequencing to search for pathogenic germline mutations might well be justified. This enables possible determination of a causative mutation in the minor candidate gene not described earlier or identification of combinations of germline mutations in several of these genes in families with a history of renal carcinoma. For example, sequencing of an exome from siblings in a family with papillary thyroid cancer and clear cell RCC revealed a combination of the *SDHA* heterozygous mutation along with mutations in the *TGFB2* and *PARP1* genes [58]. In another family, hereditary renal carcinoma with areas of angioleiomyomatous stroma was described as an atypical variant of the manifestation of the *TSC2* missense mutation [59]. Therefore, preliminary diagnosis and selection of candidate genes to search for mutations depend on the classical or, by contrast, atypical manifestation of hereditary RCC. This, in turn, determines the choice of a multigene panel for NGS, exome sequencing, and, in rare cases, traditional methods for the identification of point mutations and gross deletions.

## 4. Molecular Genetic Diagnostics of Hereditary Prostate Cancer

PC manifests as a hereditary cancer syndrome due to germline mutations in only 1% to 2% of cases. Carriers of *BRCA1* and *BRCA2* mutations are at high risk of developing PC, with *BRCA2* mutations contributing more to hereditary PC than *BRCA1* mutations [60, 61]. Lynch syndrome (incidence, 1 in 370–1,500 people in European and American populations), which is caused by mutations in the DNA-mismatch repair (MMR) genes *MLH1*, *MSH2*, *MSH6*, and *PMS2*, manifests with early-onset PC in some cases. In contrast to the manifestation of the classical form of Lynch syndrome as a hereditary nonpolyposis colorectal cancer, in hereditary PC, germline mutations are observed mainly in the *MSH2* and *MSH6* genes and much less often in *MLH1*. The proportion of *MSH2* mutations in such patients is up to 79% of cases. However, PC is not among the frequent tumors observed in Lynch syndrome (among 6,350 carriers of MMR gene mutations in a mixed cohort with an approximately equal ratio of both sexes, PC was diagnosed in 26 men). The risk of developing this cancer due to MMR gene mutations is only three- to five-fold higher, and often the first clinical manifestation of the disease and the cause of death are related to colorectal cancer, which can lead to an underestimation of the incidence of PC in patients carrying germline mutations in *MSH2/6* [62–64]. In addition to the MMR genes, the c.251G > A (p.G84E) mutation in *HOXB13* associated with a 20-fold increase in the relative risk for PC [65]. Other genes that are considered associated with hereditary PC include *TP53*, *NBN*, *BRIP1*, and other DNA-repair genes [66]. In general, according to the recommendations of the National Comprehensive Cancer Network (NCCN; v.2.2019), it is preferable to use NGS to sequence the multigene panel, which includes at least MMR genes mutated in Lynch syndrome and a number of other DNA-repair genes (*BRCA2*, *BRCA1*, *ATM*, *CHEK2*, *PALB2*, *MLH1*, *MSH2*, *MSH6*, and *PMS2*). This panel can be supplemented with other candidate genes [67]. For example, sequencing of 94 oncogenes and tumor suppressor genes was performed in 121 cases with early PC onset or a family history of the disease, resulting in pathogenic and likely pathogenic genetic variants observed in 14% of the cases. Thirteen patients harbored mutations in genes known to be candidates for initiating hereditary PC, whereas the others carried single mutations of genes not previously considered candidates for hereditary PC [68]. If minor candidate genes are excluded, then data from related studies indicate that 8% of young PC patients have hereditary PC and that a diagnostic panel for hereditary PC should include at least *BRCA1*, *BRCA2*, *ATM*, *BRIP1*, *CHEK2*, *NBN* (c.657del5), *HOXB13* (p.G84E), *MLH1*, *MSH2*, *MSH6*, and *PMS2* [69].

The categories of patients to whom molecular genetic testing should be recommended if hereditary PC is suspected were defined in recommendations made in the previous 3 to 5 years. In particular, criteria from a Johns Hopkins group included at least three first-line relatives with PC or cases of PC in three generations, as well as two relatives with PC and ≤55 years of age. The indications for testing provided by the American College of Medical Genetics in the first paragraph coincided with those from the Johns Hopkins group (three or more first-line relatives of patients with PC) and included the following assignments to the risk group: people with two or more first-degree relatives who were diagnosed with PC and ≤55 years of age; and PC with a Gleason score >7 and at least two relatives with breast, ovarian, or pancreatic cancer. Additionally, the Gleason score was considered significant starting with the previous version of the recommendations of the NCCN, which included three criteria for testing advisability: (1) PC with Gleason score ≥7 and at least one close relative ≤50 years of age with BC and/or invasive ovarian cancer; (2) PC with a Gleason score ≥7 and relatives with PC also with a Gleason score ≥7, BC, and/or pancreatic cancer at any age; and (3) patients with primary metastatic PC [69, 70]. Among patients with primary aggressive tumors, as well as those with castration-resistant PC, pathogenic germline variants are more likely to be found than on average in PC, which explains the association with initially high Gleason scores. Nevertheless, the principal criteria for diagnosing hereditary PC remain as follows: PC at a younger age (up to 55 years), family members with confirmed germline *BRCA1* and *BRCA2* mutations, or presenting signs of hereditary cancer syndromes associated with mutations in DNA-repair genes [70–72]. In the current version of the NCCN recommendations (v.2.2019), the following indications for testing are defined: patients with a family history of the disease (consonant with the 2018 version and with the addition of specific signs of hereditary cancer syndromes), intraductal histological type PC, intermediate- or high-risk primary PC, locally advanced or metastatic primary PC, and ethnic background as Ashkenazi Jew [67]. In the latter case, the recommendations refer to a population with major mutations. Summarizing these criteria, most authors suggest a search for germline mutations in patients with PC and one of the following criteria: age <55 years; immediate relatives with PC; family history of already identified *BRCA1/2* mutations or breast, ovarian, or pancreatic cancer; and PC with Gleason score >7 in the presence of signs of hereditary PC according to NCCN guidelines [70].

There might be exceptions to this approach for certain populations, in which several major mutations are present due to the founder effect. In these cases, NGS diagnosis can be performed for patients with a negative PCR test for major mutations. In particular, this applies to Ashkenazi Jews, who have three major mutations: c.68_69del, c.5266dupC (*BRCA1*), and c.5946del (*BRCA2*). The proportion of their carriers among all tested patients is ∼20%. In patients without major mutations, NGS can detect the pathogenic *BRCA1* and *BRCA2* mutation in another 8% of cases [73, 74]. Another example is the increased frequency of the c.5266dupC mutation (referred to as 5382insC in the cited publications) in the *BRCA1* gene in European part of Russia, which is responsible for 10% to 17% of cases of breast and ovarian cancer [75, 76]. The same phenomenon is also observed in some Eastern European countries with a predominantly Slavic population neighboring Russia, in particular in Ukraine [77] and Belarus [78]. Comparing the cost of two-stage testing in these populations against a general NGS-based approach, the cost of targeted NGS panels for the diagnosis of hereditary PC per analysis for one patient ranges from $250 to $1,500, depending on how many loci are included in the panel in addition to *BRCA1/2* [69]. Because the proportion of the 5382insC mutation among patients in East Slavic populations can exceed 50%, a simple and cheap (<$100) test based on real-time PCR would allow determination of the major *BRCA1* mutation in half of the tested patients within 1 day [79, 80] (unfortunately, a significant proportion of publications on this subject do not have an English version). However, we emphasize that such a two-stage search for a pathogenic mutation in the *BRCA1/2* genes can be performed only with sufficient clinical, genealogical, and population justification. In general, the main strategy for mutation search remains the use of NGS for sequencing the coding parts of hereditary PC candidate genes.

Identification of the germline *BRCA1/2* mutation has not only diagnostic but also prognostic significance. Patients with *BRCA1/2* mutations (germline or somatic) respond better to therapy with drugs that inhibit the remaining alternative DNA-repair pathway involving PARP inhibitors, as well as with platinum-based anticancer drugs. In particular, 88% of patients with *BRCA1/2* mutations respond to olaparib PARP inhibitor therapy versus 6% among those without identified mutations. The PD-1 inhibitor pembrolizumab, an immune-checkpoint inhibitor, is used for cases with severe microsatellite instability (MSI), regardless of the type of tumor, and might be prescribed to patients with Lynch syndrome [81, 82].

## 5. Lynch Syndrome as a Form of Hereditary Bladder Cancer

In the majority of cases, diagnosis of hereditary BC is reduced to diagnosing urothelial carcinoma as a part of the clinical picture of Lynch syndrome. Lynch syndrome is a hereditary cancer syndrome that usually manifests at a younger age as nonpolyposis colorectal cancer. For example, urothelial carcinoma, which is the second most frequent minor tumor after endometrioid carcinoma, has been detected in 75 of 1,624 Lynch syndrome patients [83]. As noted, Lynch syndrome is caused by germline inactivating mutations of tumor suppressor genes encoding the main components of the MMR system. The frequencies of mutations of these genes in total in all clinical forms of Lynch syndrome are 35% to 45% (*MLH1* and *MSH2*), 5% to 10% (*MSH6*), and ∼5% (*PMS2*). Detection of MSI in the tumor is used as a relatively simple screening test to genetically diagnose Lynch syndrome. Another screening test is IHC analysis with an antibody panel that can detect the lack of expression of DNA-repair factors MLH1, MSH2, MSH6, and PMS2. Concordance of results of the molecular genetic MSI test and IHC detection of these factors reaches 94%, which allows their consideration as almost equivalently effective methods for the screening of Lynch syndrome [84]. MSI and IHC have their own peculiarities of practical application. In particular, a positive MSI result in the diagnosis of Lynch syndrome is established when >30% of the studied short tandem repeat markers show the presence of aberrant alleles (MSI-High), whereas the absence of aberrant alleles (MSI-Low) is considered a negative result. The number of identified unstable loci depends on the panel of microsatellites; therefore, it is recommended to use mononucleotide rather than dinucleotide markers and at least five loci. IHC enables the identification of which repair factor is absent; however, the problem of evaluating samples with an intermediate level of staining remains. Therefore, the choice of MSI versus IHC depends more upon which method is more routine in the laboratory: PCR and fragment analysis on a capillary genetic analyzer or a stream of IHC tests using automated systems, such as Ventana [85, 86]. It should be kept in mind that both MSI and IHC are only screening tests for Lynch syndrome and that germline mutation testing is still required to confirm the diagnosis.

Mutations of *MSH2* are four-fold more frequent in Lynch syndrome with BC than mutations of *MLH1*, the candidate gene most often affected in Lynch syndrome (up to 20% of patients harboring *MSH2* mutations have BC) [63, 87]. NGS enables a pathological mutation to be identified by sequencing the coding regions of candidate genes for Lynch syndrome and other hereditary BC forms using comprehensive cancer gene panels or small customized panels designed for the disease. The fact that *MSH2* mutations are more frequent than mutations of other genes in BC might be important for selecting a less expensive NGS protocol and using panels for targeted candidate gene sequencing in Lynch syndrome [88].

## 6. Panels for NGS-Based Diagnostics of Hereditary Forms of Urological Cancers

**Figure 1:**
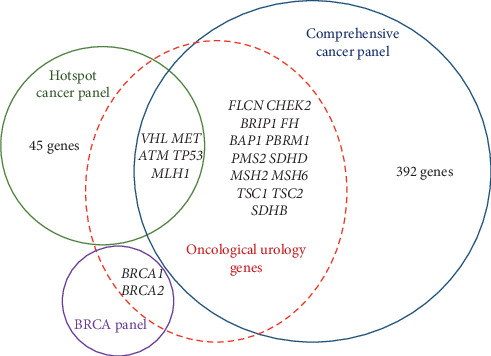
Major candidate genes for hereditary urological cancer disorders in ready-to-use AmpliSeq panels for targeted sequencing. The Venn diagram shows differences and similarities in various AmpliSeq panels. The blue circle includes genes comprising the comprehensive cancer panel, the green circle includes genes in the cancer hotspot panel, and the purple circle represents the BRCA panel. The dashed red boundary surrounds the gene mutations responsible for the development of the most common hereditary urological cancer diseases.

Patients with some of the urological cancer syndromes would benefit from an NGS-based search for causative germline mutations. The first variant of an NGS-based approach utilizes gene panels selected via a literature survey of candidate genes for hereditary urological cancers. For example, sequencing of a panel of 23 genes in young RCC patients revealed pathogenic germline variants in 10 genes in 9.5% of the patients [6]. Another study used the targeted panel designed for hereditary RCC, which includes 19 genes: *BAP1*, *FH*, *FLCN*, MET, *MLH1*, *MSH2*, *MSH6*, *MITF*, *PMS2*, *EPCAM*, *PTEN*, *SDHA*, *SDHB*, *SDHC*, *SDHD*, *TP53*, *TSC1*, *TSC2*, and *VHL*. Pathogenic genetic variants were identified in 6.1% of 1,235 patients with suspected hereditary cancers of various organs, and among the examined cases, 43.7% with available submitted histology were consistent with published literature based on the specific gene alteration. Moreover, for patients who provided sufficient personal and family history, only 32.9% had a strong suspicion for the identified gene alteration. This suggests that a significant proportion of cases with nonclassical manifestation of hereditary RCC might be false negative according to the results of the study of only one candidate gene [89]. Another approach is the use of premade panels of major oncogenes and tumor suppressor genes involved in the carcinogenesis of various tumors and offered as ready-to-use solutions by major manufacturers of equipment and reagents for NGS. Small 50-gene and an extended AmpliSeq panel of 409 genes are offered by Thermo Fisher Scientific and Illumina [90, 91]. Premade panels have the advantage of being reliable and available but are designed to test for a broad spectrum of common hereditary cancer syndromes and not specific for hereditary urological cancers. The total gene list contains only the 20 genes listed here as the main candidates for initiating hereditary urological oncology diseases (see Figure 1). Evidently and for the purposes of urological oncology, there are 45 unnecessary genes in the small panel and 392 in the large one. These findings suggest the use of multigene panels designed specifically for the diagnosis of hereditary urological cancer syndromes as the most effective diagnostic NGS approach.

Analysis of germline genetic variants obtained by NGS inevitably encounters the following issue: apart from mutations with proven clinical significance and likely pathogenic variants, as well as those that are benign or likely benign, there exists a set of variants with uncertain clinical significance (VUS) [92]. If a VUS is detected as a result of analyzing sequencing data obtained for a patient with suspected hereditary RC, PC, or BC and in the absence of pathogenic/likely pathogenic mutations, this would require a more in-depth analysis and a balanced approach to medical genetic counselling [93]. In general, primary analysis of genetic variants detected by sequencing, in particular, of AmpliSeq panels on the Ion Torrent platform is carried out using Torrent Variant Caller. For visual data analysis and manual filtration of artifacts, the Integrative Genomics Viewer (http://software.broadinstitute.org/software/igv/) is used, and identified genetic variants are annotated using ANNOVAR software (https://annovar.openbioinformatics.org/en/latest/user-guide/download/). For variant annotation, databases of human germline mutations and polymorphisms (i.e., ClinVar, HGMD, and LOVD) are used, as well as databases focused on a specific disease/gene (i.e., Consortium of Investigators of Modifiers of BRCA1/2-CIMBA). Significant factors in the analysis of pathogenicity include mutant allele frequency (MAF) and the conservatism of the variable codon. A MAF <0.01 and the presence of an amino acid change occurring in a highly conserved site represent a pathogenicity factor in the identified genetic variant [94, 95]. If there is no information on the role of a germline VUS in carcinogenesis, it is possible that data on identical somatic driver mutations are available in the COSMIC database and the Cancer Genome Atlas [96]. As a rule, VUSs include missense variants, deletions/insertions without frameshifts, and nucleotide substitutions at splice sites, with the majority representing missense variants [97]. Furthermore, VUSs can be analyzed both *in silico* and *in vitro*.

In the first case, the effect of a mutation on protein function and prediction of the pathogenicity of novel missense mutations can be evaluated using PolyPhen2, MutationTaster, SIFT, and other predictors [98]. Collation with clinical data and the results of other laboratory tests can also help reclassify VUSs. For example, in the diagnosis of Lynch syndrome, co-segregation with colorectal cancer in the pedigree and MSI-High status of the tumor of the proband indicate the pathogenic nature of the detected genetic variant [97]. In the second case, with appropriate laboratory facilities available, functional tests are used to analyze the pathogenicity of the novel mutation. In particular, for VUS analysis in *BRCA1*, the homology-directed recombination assay is used. In this approach, the VUS is inserted into the expressed vector introduced into HeLa-DR cells, and the expression of endogenous *BRCA1* is inhibited by small-interfering RNA. These cells are then transfected with a vector expressing an endonuclease that introduces DNA breaks. If the VUS is pathogenic, the cells will not be able to synthesize functionally active BRCA1 and perform effective DNA repair. As a result, cells undergo apoptosis and exhibit decreased fluorescence [99]. Functional tests also include the methylation tolerance assay, which allows reclassification of novel alleles in *MSH2* and *MLH1*, where the proportion of VUSs in patients with suspected Lynch syndrome can reach 30%. This allows expression of the allele in question in *MSH2-* or *MLH1*-deficient cell lines along with subsequent exposure to methylating agents. Normally, activity of MMR proteins should promote the death of cells harboring multiple modified noncomplementary bases; therefore, a significant percentage of surviving cells indicates impaired *MSH2*/*MLH1* function and VUS pathogenicity [100]. If tumor material is available for laboratory analysis and the VUS is localized in the tumor suppressor gene, the selective deletion of the reference homologous allele in the tumor DNA can be considered a loss of heterozygosity according to the Knudsen theory. This allows the reclassification of the VUS, thereby increasing pathogenicity score (as shown by the example involving the *BRCA2* gene) [101].

The abundance of VUSs detected within multigene panels and their even greater number in exome-sequencing output have hampered the use of genome or exome sequencing as a diagnostic tool in clinical practice, despite the examples of mutation detection by whole-exome sequencing. Errors in interpreting genetic variants are predominantly associated with VUSs (*p* < 0.001, comparing the five classes of genetic variants) [93]. Additionally, the fraction of VUSs is higher in the populations of Africa, Latin America, Asia, and Oceania than in Caucasians, for whom more results of genetic studies have been published [102]. Moreover, the fraction of VUSs among all identified germline variants can be quite high for multigene panels (i.e., 37% for a panel of 12 candidate genes for hereditary PC) [71]. Furthermore, *in silico* testing is limited, and highly reliable functional tests are often time consuming and laborious, thereby limiting their use for molecular genetic testing in oncology. Until recently, the main NGS approach remained the sequencing of gene panels with an increasing number of published approaches that allow reclassification of VUSs in the diagnosis of hereditary RCC, PC, and BC.

## 7. Conclusions

Urological oncology disorders caused by germline mutations constitute a broad set of oncology diseases, some of which are highly heterogeneous both clinically and genetically. Some of these are caused by mutations of one candidate gene and/or exhibit specific characteristics, including nonurological manifestations (VHL syndrome, BHDS, HLRCC, and HPRC), whereas other hereditary cancer syndromes are due to mutations that might affect any site in the coding region of one candidate gene (BAP1-TPDS) or one of the several genes (tuberous sclerosis, hereditary PC with *BRCA1/2* mutations, and Lynch syndrome with BC) consisting of >10 exons. In such cases, it is necessary to sequence the coding regions of all candidate genes. Premade target sequencing cancer panels include genes that are not directly related to hereditary urological cancer syndromes, and such genes account for 90% to 96% of all panel genes. In general, it is possible to formulate the main diagnostic approaches to identifying a pathogenic germline mutation that causes hereditary RCC, PC, and BC. In rare cases of the classic manifestation of a monogenic disease and/or the existence of major mutations in the population (VHL syndrome with a candidate gene of three short exons, *BRCA1/2* mutations in Ashkenazi Jews and Eastern Slavs, and an MSI test for Lynch syndrome), analysis is possible, at least in the first stage, using classical methods, such as PCR, Sanger sequencing, and MLPA. All other situations require the use of NGS. In these cases, targeted sequencing of the coding regions of tens of known candidate genes for hereditary oncological diseases is effective. The first approach with NGS is the sequencing of precast panels comprising from several tens to a broad panel of genes known to cause hereditary cancer syndromes. An advantage of this approach is the sequencing of most candidate genes for hereditary cancer syndromes, the constantly decreasing cost of the test, and the reliability of the analytical process in genetic laboratories. The disadvantages are a large number of VUSs and, consequently, difficulties in further medical and genetic counselling. Moreover, not all candidate genes for hereditary oncological diseases are included in the NGS panels. An alternative approach would be to use limited panels that include a couple of dozen genes. The results of sequencing such a panel would be easier to annotate and interpret for counselling, especially if the laboratory and the consulting doctors are focused on the diagnosis of hereditary cancer syndromes. However, there is a risk of omitting a pathogenic mutation in a minor candidate gene not included in such a limited panel. Therefore, the sequencing of multigene panels appears to be the basic solution for molecular genetic diagnosis of hereditary urological cancers.

## Figures and Tables

**Table 1:** Main hereditary urological cancer syndromes due to germline mutations.

Disorder (incidence)	Gene	Tumor type
*Renal cell cancer*		
Von Hippel–Lindau syndrome (1 : 40,000)	*VHL*	ccRCC
Birt–Hogg–Dube syndrome (n/a)	*FLCN*	pRCC, chRCC, OC
HPRC (n/a)	*MET*	pRCC type I
HLRCC (n/a)	*FH*	pRCC type II
BAP1-TPDS (n/a)	*BAP1*	ccRCC
Hereditary paraganglioma (1 : 1,200,000)	*SDHA/B/C/D/AF2*	pRCC, ccRCC
Tuberous sclerosis (1 : 6,000–10,000)	*TSC1/2*	AML
Other monogenic forms of RCC (n/a)	*PBRM1*, *FHIT*:*RNF139*	ccRCC

*Prostate cancer*		
Lynch syndrome in men with PC (n/a)	*MLH1*, *MSH2*, *MSH6*, *PMS2*	AC
Hereditary male breast cancer/PC (n/a)	*BRCA1/2*, *CHEK2*, *ATM*	AC

*Bladder cancer*		
Lynch syndrome (on average, 1 : 1,000)	*MLH1*, *MSH2*, *MSH6*, *PMS2*	UC

Abbreviations: ccRCC, clear cell renal cell carcinoma; pRCC, papillary renal cell carcinoma; chRCC, chromophobe renal cell carcinoma; OC, oncocytoma; AML, angiomyolipoma; AC, adenocarcinoma; UC, urothelial carcinoma; HPRC, hereditary papillary renal carcinoma type 1; HLRCC, hereditary leiomyomatosis and renal cell carcinoma; BAP1-TPDS, BAP1 tumor predisposition syndrome; n/a, not available.
